# Techno-economic analysis of torrefied fuel pellet production from agricultural residue via integrated torrefaction and pelletization process

**DOI:** 10.1016/j.heliyon.2023.e16359

**Published:** 2023-05-16

**Authors:** Tumpa R. Sarker, Castaneda S. German, Venu Babu Borugadda, Venkatesh Meda, Ajay K. Dalai

**Affiliations:** a Department of Chemical and Biological Engineering, University of Saskatchewan, Saskatoon, Saskatchewan, Canada; b Department of Farm Power and Machinery, Bangladesh Agricultural University, Mymensingh, Bangladesh

**Keywords:** Canola residue, Torrefied pellet, Techno-economic analysis, Break-even analysis, Sensitivity analysis

## Abstract

Torrefied pellets have gained more commercial importance due to their excellent performance in combustion, co-firing and gasification. The present investigation provides a conceptual design for torrefied fuel pellets production via combined torrefaction and pelletization technologies with and without additives. The entire design contains torrefaction unit, grinding, preparation of pellet formulation, pelletizing, and finally cooling of pellets. Two scenarios, scenario 1 (pelletization of torrefied biomass with additives) and scenario 2 (pelletization of torrefied biomass without any external additives) were tested and compared. The economic analysis suggests that both scenarios are profitable. Both scenarios were simulated using Aspen plus™, and economic feasibility was estimated using a complete cash flow analysis for a base case plant with 40,080 tonne/y capacity. For both cases, a discounted cash flow is a useful tool for estimating the minimal selling price for torrefied pellets as well as the capital investment, production cost and operating costs. The cost of the reactor used for torrefaction was found to be the most important component of combined torrefaction and pelletization system. The lowest selling price of generated torrefied pellets was found to be $103.4 and $105.1 per tonne at the plant gate for scenarios 1 and 2, respectively. Sensitivity analysis shows that, among all variable costs, labor cost has the highest influence on both net present value (NPV) and minimum selling price (MSP) in making pellets for both the scenarios. Furthermore, the internal rate of return was found to be25% and 22% at 10% discounted cash flow rate for scenarios 1 and 2, respectively. The framework that was created was found to lessen over-dependence on wood or fossil fuels and facilitate the promotion of bioenergy in rural areas.

## List of abbreviations

CAPEX: Capital expenditure
CEPCI: Chemical engineering plant cost index
DCFR: Discounted cash flow rate of return
IRR: Internal rate of return
LC: Labor cost
L_C_: Land cost
Mg: Mega gram
MSP: Minimum selling price
NPV: Net present value
OPEX: Operating expenditure
PBP: Payback period
PC: Purchase cost of Equipment
SC: Start-up cost
TCI: Total capital investments
TEA: Techno-economic analysis
TFC: Total fixed cost
TOP: Torrefaction before pelletization
TPDC: Total plant direct cost
TPIC: Total plant indirect cost
WC: Working capital

## 1 Introduction

Fossil fuels have long been regarded as unfriendly to the environment because they emit a lot of greenhouse gases (GHGs), which cause global warming. An increase of 80% in fossil fuel usage will result in a 70% increase in GHG emissions [1]. This could have a significant global environmental impact. All of these factors stimulated to focus on renewable energy sources, with biomass-based energy production playing an important role. Biomass fuel pellets are one of the major sources of energy that can be a promising substitute of fossil-based fuel especially coal [2].

The worldwide demand for wood pellets used for heating and power generation is increasing consistently. It is projected that the global demand for wood pellets will reach 52 million metric tonnes (MMT) by the year 2025, which is almost 3.3 times higher relative to 2010 [3]. The European Union led the world pellet production (13 MMT) in 2020, followed by North America (11 MMT), China (10 MMT), South America (4.4 MMT) and the rest, all of this added up to about 39.2 MMT while the European Union is the highest consumer of wood pellet followed by Asia, America [4]. Wood pellets are typically used for heating and power generation both in industrial and residential sectors [5]. It has been reported that GHG emissions were reduced by 20% from 2005 to 2020 in EU due to usages of wood pellet [6]. North America is currently leading wood pellet producers throughout the world and Canada is the second largest wood pellets producers because of their plentiful biomass supply, established industry, and effective logistics [7]. The majority of the Canadian wood pellets are produced in westernmost province of Canada, particularly in British Columbia (BC) [6].

Besides woody biomass, Canada also generates huge amount of agricultural residues annually, especially in Saskatchewan, as a major agricultural province [8]. Annual canola production in Saskatchewan was 5.9 million metric tonnes in 2020, and the major byproduct of canola seeds are a combination of pods, hulls, dead seeds, which accounts for almost 10–15 wt% of total production. Production of fuel pellets from agricultural residue can be a promising approach to benefit local and national economy [9]. However, fuel pellets production from agricultural residues as well as conventional wood pellet has some challenges such as; hydrophilicity, low heating value, poor storability as well as poor grindability [10]. Combination of torrefaction pre-treatment with densification of biomass can solve above-mentioned challenges.

Torrefaction, a thermochemical pre-treatment generally was conducted in the temperature of 200–300 °C under oxygen deficient atmosphere to decompose the lignocellulosic structure by releasing the OH groups and increasing the carbon content [8,11]. It has been well established that torrefaction produces coal like material with significantly increased energy content, grindability, hydrophobicity [10,12].

Combining torrefaction with pelletization has the potential to create a fuel pellet that possesses an energy density comparable to that of coal, paving the way for its use as a substitute for coal in heat and power plants [13]. Combined torrefaction-pelletization significantly improves the physico-chemical properties of biomass [[14], [15], [16]] and this process has gained attention for both researchers, and industries for commercial scale production.

However, the production cost of fuel pellet is important to consider along with the fuel pellet quality. Literature on the techno-economic analysis (TEA) of torrefied pellet production is scarce. Uslu et al. [17], claimed that combined torrefaction and pelletization provided higher energy density and remarkably reduced storage and transportation cost. Production cost of sawdust pellet was found to be $51/t at 45 kt plant capacity. Pirraglia et al. [18], conducted economic analysis of torrefied wood pellet with addition of binder and reported the production cost of torrefied pellet was $261/metric ton at 100,000 metric tons/y plant capacity. Manouchehrinejad et al. (2021) [19] reported the minimum selling price for torrefied wood pellet is $207 per mega gram at the plant gate. Shahrukh et al. [1], studied the production cost analysis of three different biomass including forest residue, energy crop and agricultural waste and reported that cost for producing 1 kg of pellet ranges from $95–105/t for regular pellet and $146–156/t for steam treated pellet. However, the techno-economic evaluation for fuel pellets from agricultural residue have not been explored extensively, which need to be focused for commercialization potential because the composition of woody biomass is quite different from agricultural residues. Therefore, the produced pellet quality also varies with biomass type. Moreover, torrefied biomass usually needs some external binder to bind the particles together, which is also important to consider during economic analysis. Despite the extensive study into combining torrefaction and pelletization to make fuel pellets, most of them only considered technological and financial assessment. The quality of pellet and the requirement for external binding agents are all uncertain.

As far as the authors are aware, no research has been conducted on the techno-economic analysis (TEA) of producing torrefied fuel pellets from canola residue. Nevertheless, comparison of pellet production with and without external binder does not exist in the literature. This study offers a conceptual plan for the production of pellet from canola residue using two different production routes: scenario 1 (pelletization of torrefied biomass with additives) and scenario 2 (pelletization of torrefied biomass without any external additives). Thus, the aim of this research is (1) to examine the detailed techno-economics of making torrefied pellets using an integrated torrefaction and pelletization system, (2) to estimate total capital cost and operating expenditure to run the proposed process, (3) to perform a sensitivity analysis to examine the specific details of the process and cost parameters that affects the net present value (NPV) as well as minimum selling price (MSP) of torrefied wood pellets, and (4) to compare critically the results from scenarios 1 and 2. The break-even price of pellets were calculated and the calculated price were assessed with other torrefied pellet production methods. Additionally, the current research lays the groundwork for the efficient use of Saskatchewan grown agricultural residue (canola residue) in fuel pellets production.

Binders introduced to make canola residue derived fuel pellets is the new addition to the literature. The production route of scenario 2 is also a novelty of this investigation. Moreover, pellet production using microwave torrefaction with three different additives/lubricant is the main attraction of the present investigation, which is absent in literature.

## 2 Methods

### 2.1 Basis of design

The simulation as well as modeling was carried out considering canola residue as feedstock; however, it can be used for analyzing other lignocellulosic biomasses. The reason for choosing canola residue for modeling the integrated torrefaction and pelleting unit is their availability in North America. Canada produces around 20 million tonnes while the world canola production was 70 million tonnes in 2020, generating almost 10–15% of total production as residue which usually used for landfilling, burning etc. [20,21]. The simulation was developed based on the optimum conditions reported in our previous studies [20,21].

### 2.2 Process description

Two different scenarios were assessed for production of torrefied pellets. In case of scenario 1, the raw materials are canola residue collected from canola processing plant as a byproduct. The pellet production unit is assumed to be located near the canola processing plant to avoid the transportation cost. The plant is assumed to process 5 tonnes/h or 120 tonnes/day of canola residue. Since the moisture content of canola residue as received from processing plant ranges from 10 to 12 wt% (w.b), the materials were directly sent for torrefaction. Energy for torrefaction was supplied from electricity to produce microwave irradiation into the biomass materials to heat them up. After torrefaction, two different products can be obtained such as volatiles and torrefied biomass. The torrefied biomass was cooled to room temperature by blowing cold air for safe handling while the volatiles obtained from torrefaction was condensed into condensable liquid and non-condensable gases. The compositions of non-condensable gases are CO_2_, CO, CH_4_ while the liquid products contained water, organic acids, phenols, furfural, etc. The size of torrefied solid products was reduced using knife mill at 1.7 mm screen size and the pulverized ground biomass was used for pelletization. Prior to pelletization, the ground torrefied biomass was mixed with different bio-additives to form pellet formulation. Then, the pellets were produced from the pellet formulation and cooled immediately to room temperature to avoid the heat stress. Upon cooling, the pellets were stored in silo for packaging and distribution.

The plant was designed for optimum torrefaction operating conditions (microwave power: 250 W, temperature: 178 °C and residence time of 10 min). The additives concentration was maintained at 10 wt% of alkali lignin, 10 wt% of mustard meal and 15 wt% of pyrolysis oil [9].

In scenario 2, condensable liquid obtained from torrefaction was used as lubricant during pellet formulation. The concentration of torrefied liquid used was 15 wt% and water was sprayed to get the moisture content of the mixer as 12%. In scenario 2, no external additives were used to enhance pellet quality and the process was identical to scenario 1.

### 2.3 Process simulation model

Aspen Plus v12 (Aspen Tech, Bedford, USA) was used to model and simulate the integrated torrefaction and densification process for the generation of torrefied fuel pellets from canola residue. The process started with collection of biomasses from canola processing plant in western Canada. Then, the canola residue was torrefied under microwave irradiation at microwave power 250 W for 10 min at Rstoich reactor (TORREC) which is considered as a reactor for torrefaction. During torrefaction, energy was supplied by electricity to heat the biomass. In that case, a nitrogen atmosphere was created to ensure that the reaction happens under inert gas by supplying nitrogen flowing at 100 ml/min. The simulation model was utilized for each configuration to calculate the energy and mass necessary for a plant operating at the base capacity of 40,080 tonne/y, as well as to estimate the size of each unit operation required for overall techno-economic analysis. There is no built-in torrefaction model in Aspen Plus™ software, therefore, Rstoich reactor was used. The products of torrefaction are the solid products (torrefied biomass), condensable liquid and non-condensable gases. Torrefied biomass was conveyed to GRINDER by a screw conveyor (SCRW-CON) where the temperature of torrefied biomass reduced by blowing air. Size of torrefied biomass was reduced using an impact mill (GRINDER) at screen size of 1.7 mm. Then, the ground torrefied biomass was mixed with different biobased additives in a mixer (MIXER) in order to formulate the pellet formulation, which were fed to the pelletizer (GRANULATOR). Aspen Plus doesn't have any extruder or pelletizer, therefore, granulator was used to represent pelletizer. Then, the produced pellet was sent to storage via conveyor (PELCON) where pellet was cooled down at room temperature. On the contrary, the condensable liquid and non-condensable gases (C-NCGAS) was condensed in a condenser at 25 °C and separated into gases (OFFGAS), liquid products (TORLIQ) and water (WASWATER). Table S1 represents the list of all of the processes that were used in the simulation, as well as their specifications.

Here, two different scenarios were compared. First one considered pelletization of torrefied biomass where different bio-based additives were applied to improve pellet quality while the torrefied liquid can be sold as by-product. In the second scenario, torrefied liquid was used as additives for pelletizing torrefied biomass. For conditioning, part of wastewater was also used during mixing for preparing pellet formulation.

**Fig. 1:**
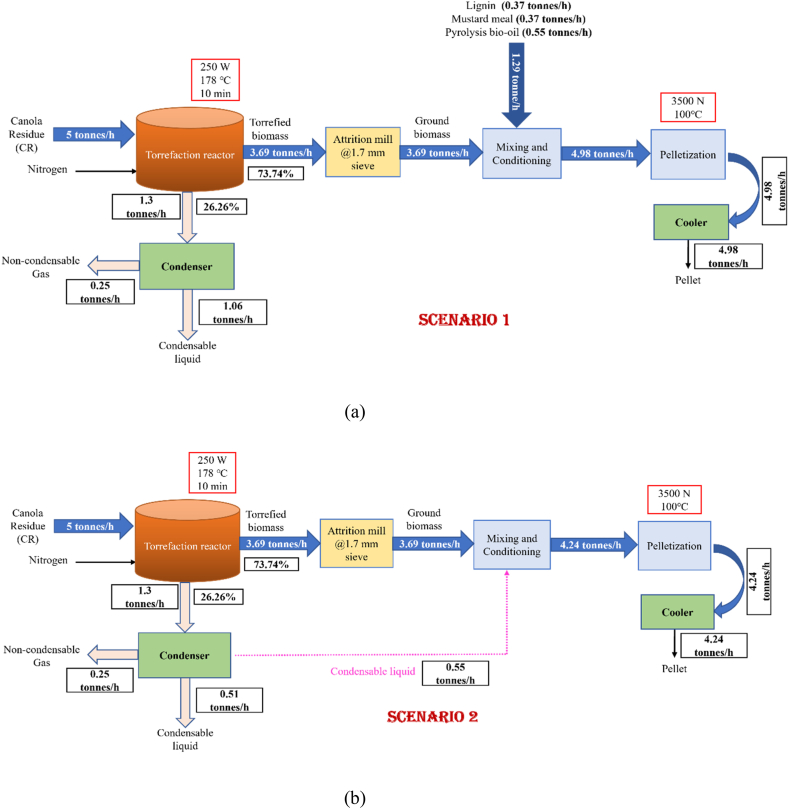
Block diagram and overall mass balance for designing a process for production of torrefied fuel pellet from canola residues.

Additionally, the components of canola residue were determined based on ultimate and proximate analysis which was used to define canola residue as non-conventional components during simulation. Canola residue contains 46.1 wt% of C, 5.7 wt% of H_2_, 2.2 wt% of N, 0.7 wt% of S and 37.7 wt% of O. Moreover, the moisture, ash, volatile matter as well as fixed carbon contents of canola residue were found to be 8.9, 75.9, 7.3, 7.9 wt% (db), respectively. The overall process was described in a block diagram for two different scenarios as shown in Fig. 1 where Fig. 1(a) represents addition of bio-additives for pelletization and Fig. 1(b) depicts the pelletization of torrefied biomass without any external binders. The Aspen Plus simulation of two scenarios are presented as Figs. S1 and S2.

In this simulation, the PR-BM global property technique was employed to estimate the properties of the non-traditional components involved in the process of torrefaction and pelletization. The Peng Robinso-e− Boston Mathias property method was used to calculate thermodynamic properties during the Aspen Plus simulation. Table 1 summarizes all the assumptions made in Aspen Plus to simulate the design and economic model.

**Table 1:** A set of assumptions applied in Aspen Plus v12 simulation and economic model.

Parameters	Assumption
For process simulation	
Biomass components	C, H_2_, N, O, S, Ash
Decomposition of biomass during torrefaction	Torrefied biomass, condensable liquid (acetic acid, furfural, water, aldehyde), non-condensable gases (CO_2_, CO, CH_4_)
Equation of state	PR-BM equation
Economic model	
Base year	2021
Plant capacity (CR)	40,080 tonne/yr
Total operating days	334 (24 h in day)
Plant lifetime	20 yrs
Plant construction duration	1 year (9 months construction and 3 months start-up)
Currency used	USD
Land Cost	2% of fixed capital investment
Depreciation	Straight line method
Depreciation period	19 years
Salvage value	10% of plant price

### 2.4 Cost estimations and equipment sizing

After simulating the conceptual design as well as mass and energy balance, it is important to calculate and sizing the equipment. The total cost of a plant includes overall investment cost and entire operational cost [22]. The details of cost calculation are described in the following sections.

#### 2.4.1 Capital expenditure (CAPEX)

Capital expenditure (CAPEX) includes both direct and indirect cost. CAPEX encompasses various costs, including Total Fixed Capital (TFC), Working Capital (WC), start-up and validation costs, up-front R&D expenses. TFC is further divided into Total Plant Direct Fixed Cost (TPDC) and Total Plant Indirect Fixed Cost (TPIC). TPDC includes the capital invested in the plant's equipment, processing, piping, instrumentation, buildings, and facilities, while TPIC covers engineering and construction costs [19]. In addition, miscellaneous costs such as contractor's fee and contingencies are also considered. However, the equipment purchases cost (PC) was calculated based on the equipment size and capacity of plant using (Chemical engineering guideline). PC also known as bare module cost as same method used for determining equipment purchase cost [23]. The parameters and assumptions used for estimating CAPEX are listed in Table 2, and all economic evaluations were conducted in US dollars in 2021. It should be noted that the equipment purchase cost is based on the 2004 CEPCI book value [21] and needs to be updated to reflect the current year. To adjust the equipment purchase cost to the year 2021, the Chemical Engineering Plant Cost Index (CEPCI) was employed using Equation (1). The plant index of 2021 is stated as 699.97 [24].

(1) $$C_{current}=C_{reference}\left(\frac{{CEPCI}_{current}}{{CEPCI}_{reference}}\right)$$

**Table 2:** Summary for calculating total capital cost (CAPEX).

Cost category	Assumptions used for estimation
Purchase price of all equipment (PC)	Equipment purchase cost
Installation cost (i)	50% of PC
Instrumentation cost (ii)	30% of PC
Cost for piping (iii)	60% of PC
Electrical facilities (iv)	20% of PC
Building and services (v)	20% of PC
Yard Improvements (vi)	5% of PC
Auxiliary facilities (vii)	40% of PC
Total plant direct cost (TPDC)	PC + i + ii + iii + iv + v + vi + vii
Engineering (viii)	12% of TPDC
Construction (ix)	10% of TPDC
Total plant indirect cost (TPIC)	viii + ix
Contractor's fee (x)	5% of (TPDC + TPIC)
Contingency and Research & development (xi)	5% of (TPDC + TPIC)
Miscellaneous (M)	x + xi
Total fixed capital cost (TFC)	TPDC + TPCI + M
Working capital (WC)	5% of TFC
Start-up cost (SC)	15% of TFC
CAPEX	TFC + WC + SC

Basic economic evaluation assumptions include 20 years project life with 9 months construction and 3 months startup period. The plant runs continuously for 24 h per day and operates for 334 days each year, giving a total of 8016 h per year.

#### 2.4.2 Operating expenditure (OPEX)

There are two types of operating cost such as fixed operating costs and variable operating costs. The fixed operating cost comprise of labor cost, maintenance cost, royalties, insurance and tax, supervision, and overhead cost [23]. On the other hand, variable operating cost consists of feedstock cost, additives cost, and cost for utility. The operating cost calculation procedure used are shown in Table 3. The labor cost is often calculated as the sum of the total number of employees each shift, total numbers of plant operating hours per year and labor wage per hour. The total numbers of operators required per shift is calculated by the following equation (Eq. 2) [25].

(2) $$N_{Ol}=\sqrt{\left(31.7P^{2}+0.23N_{NP}+6.29\right)}$$

where, N_OL_ = Total number of operators required, P=Number of solid handling steps, N_NP_= Number of on-particulate processing steps.

**Table 3:** Operating cost estimation methods.

Operating Cost Analysis	
Cost item	Assumption
Labor cost	LC
Supervision + Clerical (xii)	20% of LC
Maintenance Cost (xiii)	3% of TFC
Royalties, Insurance and Tax (xiv)	2% of TFC
Overhead Cost (xv)	50% of (xii + xiii + xiv)
Additional Expenses (Logistics, marketing, etc.) (xvi)	1% of TFC
Total fixed operating cost (TFOC)	LC + xii + xiv + xv + xvi
Cost of canola residue (xvii)	$12/tonne
Lignin cost (xviii)	$250/tonne
Pyrolysis oil cost (xix)	$0.54/gallon
Mustard meal cost (xx)	$16/tonne
Total cost of additives (A)	xviii + xix + xx
Total cost for raw materials (xxi)	xvii + A
Electricity (xxii)	$0.077/Kwh
Total variable operating cost (TVOC)	xxi + xxii
OPEX	TFOC + TVOC

#### 2.4.3 Profitability analysis and minimum selling price of pellet

Discounted cash flow analysis (DCFA) was used to estimate the minimal selling price of torrefied fuel pellet and economic viability of diverse processes. The plant is assumed to operate continuously for 24 h a day, 334 days a year, totaling 8016 h per year. The construction and startup periods were assumed to be 9 months and 3 months, respectively, with a steady production rate anticipated from the beginning of the first year until the end of the plant life, which was assumed to be 19 years. All capitals are assumed to be invested at the start of construction. The project is assumed to be 100% equity financed, with investors receiving shares of common stock rather than debt financing. The discount rate used in the analysis was based on a 100% equity structure. The salvage value and tax rate of the project was considered as 10% and 35%, respectively. The minimum selling price (MSP) was determined using a 10% discount rate, where the net present value (NPV) drops to zero after taxes.

The economic model used the principles of capital investment analysis to estimate the net present value (NPV), internal rate of return (IRR), and payback period (PBP) for a given initial capital investment to perform profitability analysis along with revenue projection [26].

NPV represents the variation between the present value of cash inflows and the present value of cash outflows from the production and sale of fuel pellets over a given period of time, taking into account the time value of money. A positive NPV designates that the investment is beneficial, while a negative NPV signifies that the investment will result in a net loss. For NPV calculation, operational costs as well as annual cash flow over 19 years pellet plant life was used, can be higher as well while the annual cash flow started in 2022 to end in 2043. NPV can also be used to calculate the capital investments' value. Moreover, internal rate of return (IRR) is an another financial metric used to estimate the profitability of a potential investment. The internal rate of return (IRR) is a discount rate that equates discounted benefits and costs simply where the NPV of an investment becomes zero [27]. A higher IRR indicates a more profitable investment. In various industries, the general rule is to determine the feasibility of a capital investment based on whether the internal rate of return (IRR) exceeds the investor's minimum acceptable rate of return or cost of capital [28]. In case of pellet plant, investors favor high IRR values that can exceed the cost of capital significantly since IRR indicates how much value can be added to the business. The payback period (PBP) is the time it requires to recoup the initial investment using profits generated from the start of a project.

The economic model was created using USD as the currency denomination and a location factor (0.91) could be used to demonstrate the cost differences between Canada and other geographics (e.g. Europe). The suggested plant is located in Canada in the province of Saskatchewan. Based on current market transportation and logistics costs, the cost of canola residue was estimated to be US $12/tonne.

#### 2.4.4 Sensitivity analysis

Sensitivity analysis is used to evaluate the influence of various factors, including fluctuations in parameters such as plant capacity, selling price of product, and capital cost on plant economics. This enables an assessment of the effect of alterations to these parameters on the base case scenario. For sensitivity analysis, few cost variables were considered such as feedstock cost, additives cost including lignin price, mustard meal price and pyrolysis oil price, pellet price, income tax, and labor cost.

## 3 Results and discussions

### 3.1 Mass and energy balance

Fig. 1 displays the mass balance for different scenarios 1–2 for torrefied fuel pellet production whereas the value was obtained from Aspen Plus simulation. They help to simplify the design of alternative processes by evaluating the flow of mass inputs and outputs. The proposed plant is designed for handling 120 tonnes/day or 5 tonnes/h of canola residue for torrefaction. After torrefaction, around 88.5 tonnes/day (3.69 tonnes/h) of torrefied biomass was produced, which was pelletized with the blending of additives at 8.8 tonnes/day (0.37 tonnes/h) of alkali lignin and mustard meal as well as 13.3 tonnes/day (0.553 tonnes/h) of pyrolysis oil. On an average 4.98 tonnes of pellet was produced per h. The condensable liquid can be used for further fuel processing but in that case, this earns by-product credit. The liquid products mainly contain water, acetic acid, furfural, aldehyde, esters etc. The amount of condensable liquid generated from torrefaction is 1.06 tonnes/h while 0.25 tonnes/h accounted to non-condensable gases. The produced flue gas from torrefaction was exhausted. It has been reported that the offgas of torrefaction can supply the energy for torrefaction via combustion [18,29]. However, this study found that combustion of offgas to generate heat is not economically feasible due to high cost of utility for combustion as well as combustion rector.

### 3.2 Economic analysis

**Fig. 2:**
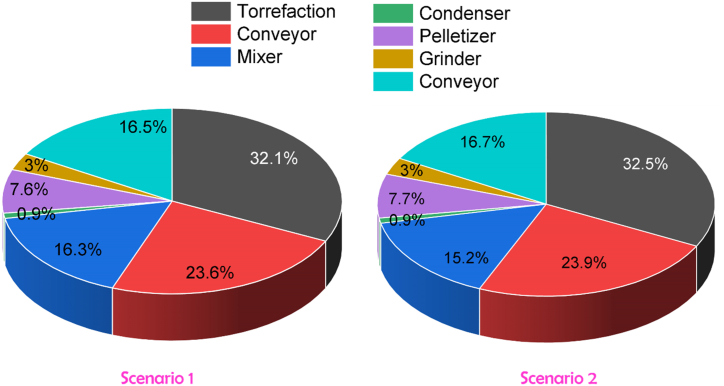
Breakdown of equipment purchase cost for two scenarios.

The breakdown of equipment purchase cost for two different scenarios is shown in Fig. 2. It can be seen that torrefaction reactor is the most expensive unit (32%) followed by the conveyor (23%) for cooling the torrefied biomass and mixer (16.3%). The pelletizer costs only 7% of overall equipment cost. The high cost in torrefaction reactor is due to its’ operation at high temperature and microwave facilities. Manouchehrinejad et al. (2021) reported the cost share for torrefaction reactor is around 34% of total capital cost (TCI) for TOP process. Another study reported that 45% of the total capital cost (TCI) for the TOP configuration came from the torrefaction reactor [30]. The cost of the conveyor is also significant, as it reduces the temperature of torrefied biomass by blowing air which then passes it to the grinder unit. Additives and ground torrefied biomass are necessary to uniformly mix prior to pelletization for ensuring strong particle bonding which cost around 16% of PC.

In case of Scenario 2, the cost of all equipment is almost same except the mixer (15%) due to less amount of material handling compared to the blending of torrefied biomass and additives. The following is the order in which the cost decreases: Scenario 1 (0.82 million USD) > Scenario 2 (0.8 million USD).

**Fig. 3:**
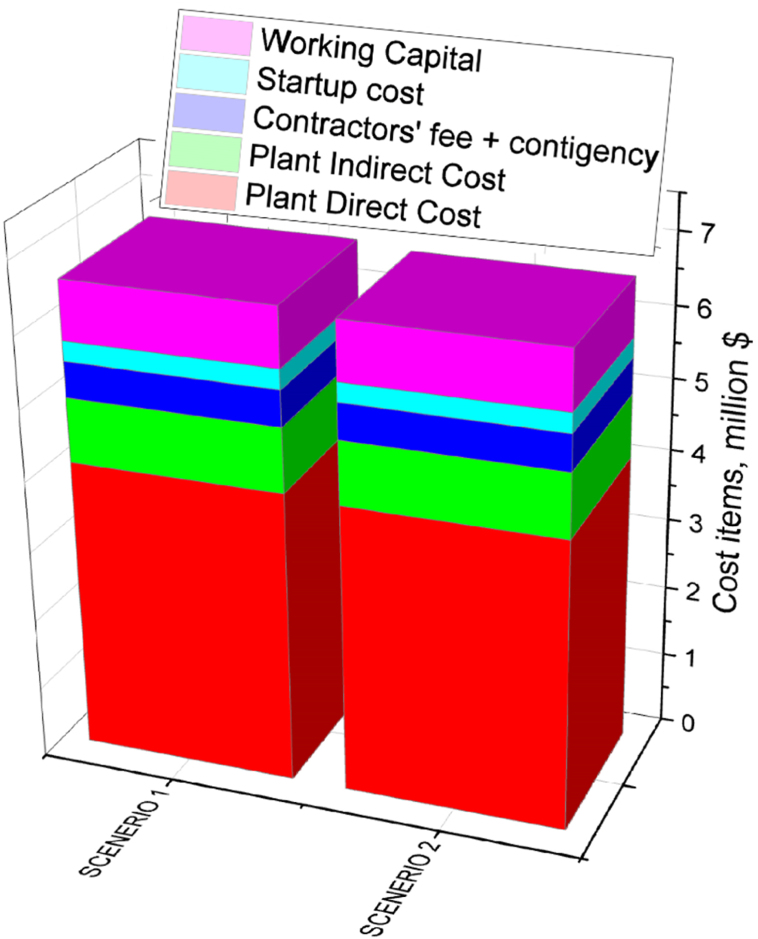
Comparison of CAPEX distribution among two scenarios for torrefied fuel pellet production.

The overall CAPEX for scenario 1 and scenario 2 was found to be 6.62 million USD and 6.54 million USD, respectively (Fig. 3). The CAPEX includes contractors’ fee, contingency which directly related to indirect plant cost. The start-up cost (SC) and working capital (WC) are also included in CAPEX which was calculated based on total fixed cost. Total fixed capital cost was found to be $5.5 million and $5.4 million for scenario 1 and scenario 2. The working capital and startup cost was 4% and 13% of total CAPEX. The capital cost of torrefied pellet plant was usually higher than conventional pellet plant due to high cost of torrefaction reactor. Similar observation was reported by Manouchehrinejad et al. [19], who found that total capital investment (TCI) required for torrefied pellet plants was 55–75% greater than that of traditional wood pellet plants.

**Fig. 4:**
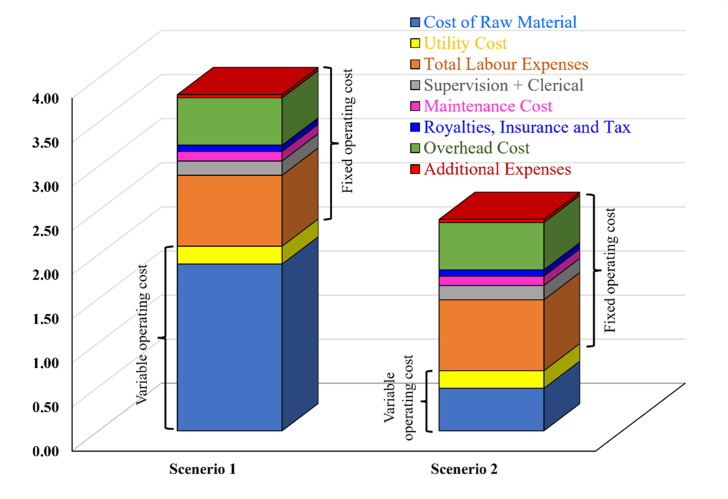
Comparison of the total operating expenses in torrefied fuel pellet plants in two different scenarios.

Fig. 4 depicts the breakdown of OPEX of two different scenarios of torrefied pellets production. The scenario of OPEX follows the trend as: scenario 1 (3.95 million USD) > scenario 2 (2.54 million USD). The greater OPEX for scenario 1 is due to higher variable operating cost including feedstocks cost, cost of additives, labor as well as utility. Usages of additives increased the total cost of raw material in scenario 1. Among all the OPEX items, raw material cost is the highest followed by labor cost for Scenario 1. Raw material cost shares 48% and 19% of overall OPEX cost for scenarios 1 and 2, respectively. The addition of lignin as binder during pelletization increased the overall raw material cost due to its high purchase price. Price of commercial pyrolysis oil also played a great role in increasing the raw material cost. All other cost items remain almost the same for both scenarios. The operating labor, overhead, and supervision costs were found to be 20%, 14%, and 4% for scenario 1 and 32%, 22% and 6% of overall operating costs for scenario 2, respectively. Utilities, maintenance and insurance including taxes and royalties account for 5%, 4% and 3% of total OPEX for scenario 1 and 8%, 6% and 4%, respectively for scenario 2. In case of Scenario 2, labor cost is the highest followed by overhead cost and raw material cost of OPEX. This project considered a total of 23 labor working in 3 shifts while each shift lasts for 8 h. The annual salary for a labor was assumed as $35,000/y [31].

In the case of OPEX, feedstock price is important, and it has to be long-term price considering the wide range of supplier price. For base case, the price of canola residue was considered as $12/tonne. The price of lignin was assumed $250/tonne [32] while mustard meal price was considered as $16/tonne. The pyrolysis oil used here is crude oil obtained just after fast pyrolysis of softwood. The oil contains around 45% water. According to literature, the pyrolysis oil price is $0.94/gallon [33]. But, here $0.54/gallon was used for pyrolysis oil as a purchasing price due to it high water content and impurities.

The main product of the plant is to produce torrefied fuel pellets. However, during torrefaction, some organic compounds are also produced as byproducts that could be sold for additional revenue. The pellet price for this study was considered as $170/tonne for torrefied canola residue pellet in both cases for easier comparison. However, the pellet quality of scenario 1 is higher than scenario 2, therefore, the cost of pellet may vary. The assumptions of pellet price were made based on literature. Agar [34], reported the market price of torrefied pellet as $151/tonne. Pirragila et al. [18] calculated the price of torrefied pellet as $260/tonne for 100,000 metric tonnes/h of plant capacity. Plant capacity largely affects the pellet price. Visser et al. [35] reported that the average cost of pellet as €136/tonne ($161/tonne) and €143/tonne ($169/tonne) for plant capacity of 500 kt/y and 50 kt/y, respectively. Mupondwa et al. (2012) [28] used an average price of $120/t for biomass pellets from wheat straw used for heat applications.

The by-products of torrefaction are condensable liquid and non-condensable gas. The gas mainly contains CO_2_ followed by CO, CH_4_. Since the liquid product of torrefaction contains large amount of acetic acid, and aldehyde, therefore, it can be used for generation of different acids. From a techno-economic standpoint, torrefaction byproducts particularly the condensable liquid, have a higher economic potential because it can be used as green chemicals for the production of acetic acid, formic acid, furfural, and methanol [36]. Torrefied liquid can also be used for production of liquid smoke.

The total annual revenue (7.7 million USD) includes total revenue from torrefied pellet (6.8 million USD) and revenue from torrefied liquid (0.9 million USD) for scenario 1. On the contrary, the total revenue from scenario 2 is 5.8 million USD. The breakdown of revenue was tabulated in Table S2.

### 3.3 Profitability analysis

Discounted cash flow analysis is commonly used to estimate the minimum price of product sell and to analyze the profitability of a process plant. By comparing net present value (NPV), payback period (PBP), and discounted cash flow rate of return (DCFR), the profitability of torrefied fuel pellet production from various routes was determined. The detailed description on profitability index analysis was reported by Javier and Ortiz, [37].

**Fig. 5:**
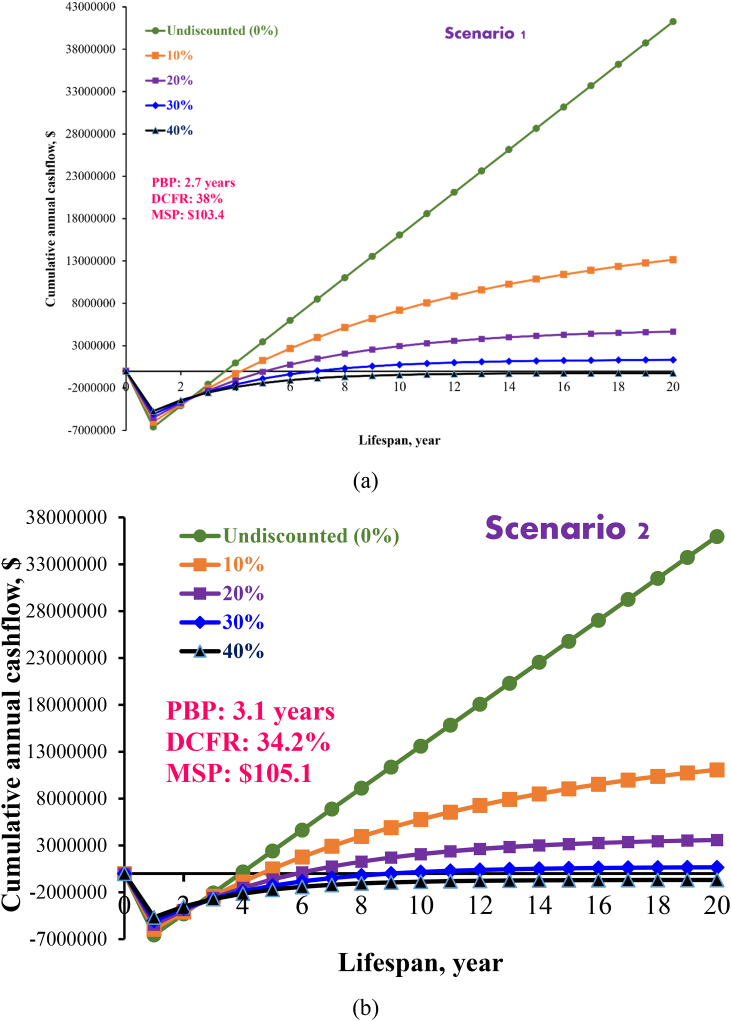
Undiscounted and discounted cash flow analysis for two scenarios of torrefied fuel pellet production.

Fig. 5 (a) and Fig. 5 (b) depicts the undiscounted and discounted cash flow for scenario 1 and 2 for torrefied fuel pellet production, respectively. Discount rates are used to normalize the value of money to the same era while taking interest rates into account. Here, the discount rate (i) varied from 0 to 40%. NPV, PBP, and DCFR, were also estimated at various discount rates. As shown in Fig. 5 (a.b), at the year zero, negative cash flow was observed because of initial capital investment and land price. Furthermore, funds are recovered from revenue when the construction is completed, and the project is launched. These funds ensure that over time, the cash flow becomes positive. The payback period (PBP), is the time required to recuperate the original investment, was 2.7 years for scenario 1 and 3.1 years for scenario 2 at the same pellet price at $170/tonne. However, the pellet quality for scenario 2 is slightly lower than that of pellet produced from scenario 1 due to poor binding mechanism of torrefied biomass. Therefore, the price of pellet should be lower than the price for pellet with additives. But, for ease of comparing two different ways for torrefied pellet production, the pellet price was kept the same. The PBP can be calculated by tracing the point on the cash flow curve when the total cash flow surpasses the level of negative working capital. Any investment must have a PBP that is lower than the duration of the project for it to be economically feasible. This study found PBP is lower for undiscounted and discounted case for both scenarios compared to the entire life of the project, therefore, it is worthy to mention that the proposed project is economically profitable for the production of torrefied fuel pellets from available canola residue. However, Payback Period (PBP) metric only reveals how long it will take to recover the initial investment and does not provide insight into the project's performance beyond that point. Therefore, Net Present Value (NPV) and Discounted Cash Flow Rate (DCFR) are additional indicators of profitability that complement the PBP.

Positive NPV was found for both scenarios which indicates the project is profitable in both ways. The highest NPV for both cases was obtained when discount rate is 0% ($41.3 million and $35.9 million for scenario 1 and scenario 2, respectively) while discounted cash flow NPV varied from $-234,374.3 at i = 40% to $13.1 million at i = 10% for scenerio 1 and $-679,974.4 (i = 40%) and $11.1 million (i = 10%). The highest DCFR was obtained at the discount rate when NPV is zero and DCFR for scenario 1 and 2 was found to be 38.0% and 34.2%, respectively. The higher the DCFR, higher the profitability of a particular project.

The scenario 1 shows the highest profitability based on the PBP, NPV and DCFR. However, the scenario 2 has lowest product output, to make this more profitable the plant capacity needs to be increased. Since the pelletizer was designed to handle 5 tonne/h but in that case only 4 tonne/h was supplied. However, additional feedstock inlet in torrefaction unit will increase the cost of torrefaction reactor and conveyer.

Though DCFR, NPV and PBP are usually used to assess the profitability of a process plant. However, DCFR still has some limitations. When comparing two projects based on DCFR, one can be tempted to select the project with a greater DCFR exclusive of considering the unique capital investment needed for each project, which changes based on its size and configuration. When comparing two projects by using DCFR, for example, one might be tempted to choose the one with a higher DCFR without taking into account the individual capital investment required for each project, which varies depending on its size and configuration. Therefore, the internal rate of return is the best profitability index, and the estimated IRR value was 25% and 22% at 10% discounted rate for scenarios 1 and 2, respectively. The positive IRR indicates that both scenarios are profitable.

The minimum selling price of torrefied pellet from canola residue was estimated for plant capacity of 120 tonne/day of feedstock processing at 10% discount rate. The minimum selling price of pellets with additives was $103.4/tonne and without additives $105.1/tonne indicating that the project only becomes lucrative when the pellets’ selling price is higher than the break-even point. The minimum selling price of produced pellets were compared with reported literature and commercial pellet cost. The MSP of torrefied wood pellet was $207/tonne of 100,000 Mg of plant capacity per year [19]. Agar [34], reported that the MSP of torrefied wood pellet is $211/Mg based on 2017 USD. Pradhan et al. [29], used $114/tonne pellet price for economic analysis of a torrefied pellet plant of 2 tonne/h capacity. Mupondwa et al. [28], demonstrated that a positive NPV can be attained for wheat straw pellet at a plant capacity of 12 ton/h if the price is greater than $100/ton. In their comparison of the costs involved in producing fuel pellets under three different conditions: conventional pellet, slightly torrefied pellet, and heavily torrefied pellet, Chai and Saffron [38], found that the slightly torrefied pellet had the lowest production costs of the three.

### 3.4 Sensitivity analysis

**Fig. 6:**
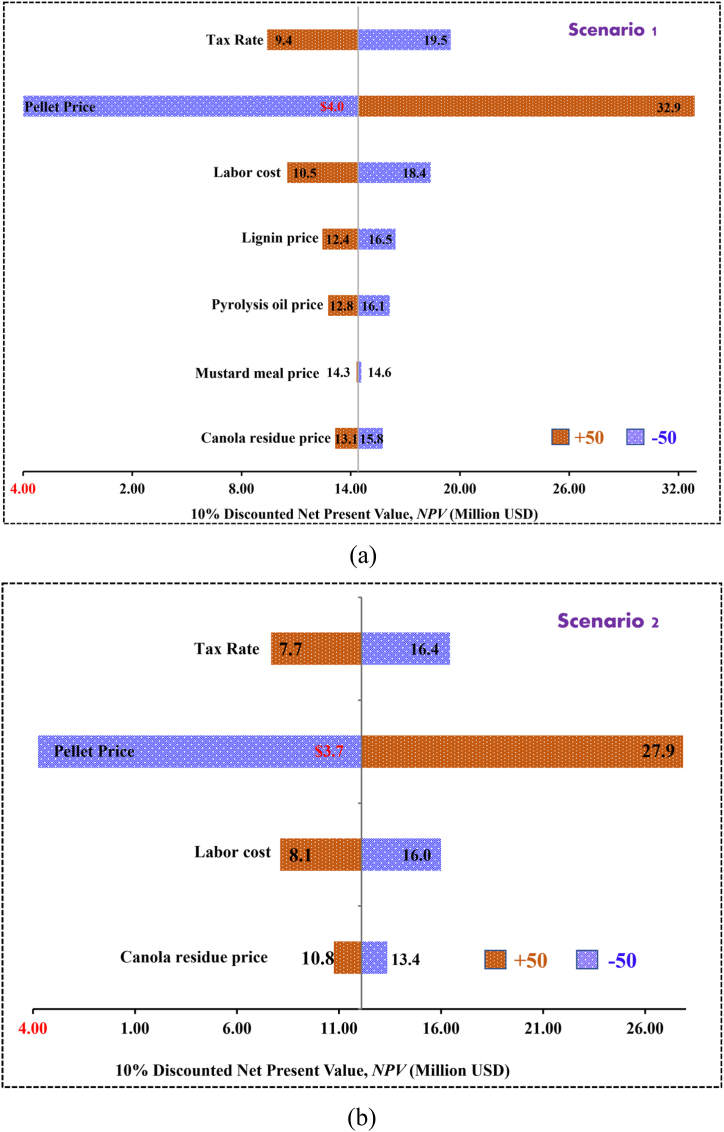
Sensitivity analysis on NPV at 10% discount rate for scenario 1 and scenario 2.

**Fig. 7:**
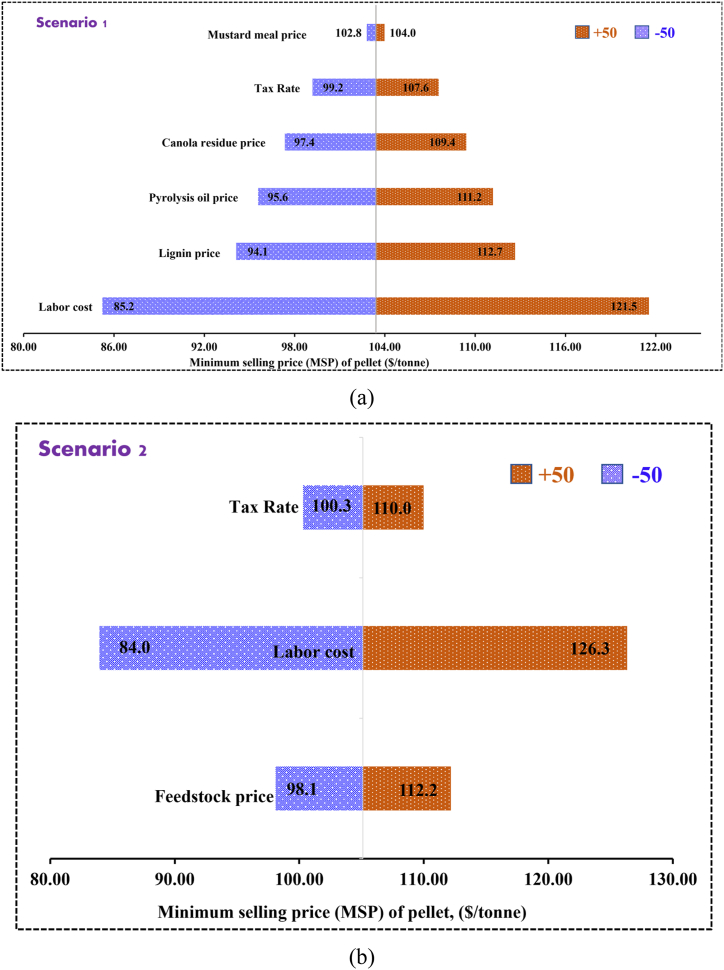
Sensitivity analysis on minimum selling price of pellet for scenario 1 and scenario 2.

The sensitivity analysis was conducted to explain how different parameters affects the NPV along with the minimum selling price of torrefied fuel pellets. These parameters include feedstock cost, pellet prices, utility cost, tax rate and cost of labor. To conduct sensitivity analysis, all these items varied to ±50. Sensitivity studies are conducted by changing one input variable while keeping the remaining variables at their nominal values. Fig. 6 (a) and Fig. b (a) show the sensitivity analysis of NPV at 10% discount rate of torrefied fuel pellet production for scenario 1 and scenario 2, respectively. On contrast, Fig. 7 (a) and Fig. 7 (b) show the sensitivity analysis of minimum selling price of torrefied pellets for scenario 1 and scenario 2, respectively. The pellet price has the highest influence on NPV for both scenarios, a 50% increase in pellet price increased the NPV from $14.4 million to $32.9 million; on the contrary, 50% reduction decreased NPV from $ 14.4 to -$4.0 million for scenario 1 while in case of scenario 2 those values varied from $12.1 to $27.9 million and $12.1 to -$3.7 million, respectively. Reduction of pellet price by 50% provides negative NPV which is not economically feasible. In case of operating cost, labor cost influenced the NPV most followed by tax rate, lignin price, pyrolysis oil price, and then raw canola residue price for scenario 1. In contrast, the second most influential factor on NPV for scenario 2 is tax rate followed by labor cost and canola residue price.

In case of minimum selling price, labor cost is the most influencing factor in both scenarios. The trend of influence of different factors on MSP of torrefied fuel pellets are as follows: labor cost > lignin price > pyrolysis oil price > canola residue price > tax rate > mustard meal price and labor cost > feedstock (canola residue) price > tax rate for scenario 1 and 2, respectively. MSP of pellet declined to $85.2/tonne for scenario 1 and $84.0/tonne for scenario 2 by 50% decrease in labor cost. Tax rate is least sensitive to MSP of pellet, a 50% increase in tax rate affect the MSP by 4%.

### 3.5 Overall discussions

The cost estimation of this process was determined based on Canadian aspects. Geopolitical projected cost or volatile in nature may vary depending on geographic material and labor. The cost of items can also vary from country to country depending on material availability, R&D price, and cost of material. The annual production cost can also be affected by market challenges, supply chain constrains, parts and services source, cost of training for labor, operator, high-quality person. The overall cost for initial investment as well as operating cost can be added by approximately 10–15% based on the above-mentioned challenges.

## 4 Conclusions

This study proposed two scenarios in producing torrefied pellets from canola residue via an integrated torrefaction and pelletization unit for processing 120 tonne feedstock per day. Both scenarios are financially feasible based on the calculated NPV, PBP and MSP determined. The highest NPV at 0% discount rate was obtained for scenario 1 ($41.3 million). The payback period of scenario 1 and 2 was found to be 2.7 and 3.1 yr, respectively. However, the minimum selling price of torrefied pellet was slightly higher in scenario 2 compared to scenario 1. Labor cost, raw material price and tax rate significantly influenced the selling price of pellet. However, more research needs to be conducted on economics and market variations of torrefied pellet production from agricultural biomass for potential commercialization aspects.

## Author contribution statement

Tumpa R. Sarker: Conceived and designed the experiments; Performed the experiments; Analyzed and interpreted the data; Contributed reagents, materials, analysis tools or data; Wrote the paper. C. S. German: Analyzed and interpreted the data; Wrote the paper. Venu B. Borugadda: Contributed reagents, materials, analysis tools or data; Wrote the paper. Venkatesh Meda, Ajay K. Dalai: Analyzed and interpreted the data; Contributed reagents, materials, analysis tools or data; Wrote the paper.

## Data availability statement

Data will be made available on request.

## Declaration of competing interest

The authors declare that they have no known competing financial interests or personal relationships that could have appeared to influence the work reported in this paper.
