# Supplementary material for: Techno-economic analysis of torrefied fuel pellet production from agricultural residue via integrated torrefaction and pelletization process
Source: Heliyon. 2023 May 16;9(6):e16359. doi: 10.1016/j.heliyon.2023.e16359 (PMC10227336; doi:10.1016/j.heliyon.2023.e16359)
Supplement: Multimedia component 1 [file mmc1.docx]

**Techno-economic analysis of torrefied fuel pellet production from agricultural residue via integrated torrefaction and pelletization process**

Tumpa R. Sarker, Castaneda S. German, Vane B. Borugadda, Venkatesh Meda, Ajay K. Dalai*

Department of Chemical and Biological Engineering, University of Saskatchewan, Saskatoon, Saskatchewan, Canada

***Corresponding author:** Dr. Ajay K. Dalai (Email: [ajay.dalai@usask.ca](mailto:ajay.dalai@usask.ca))


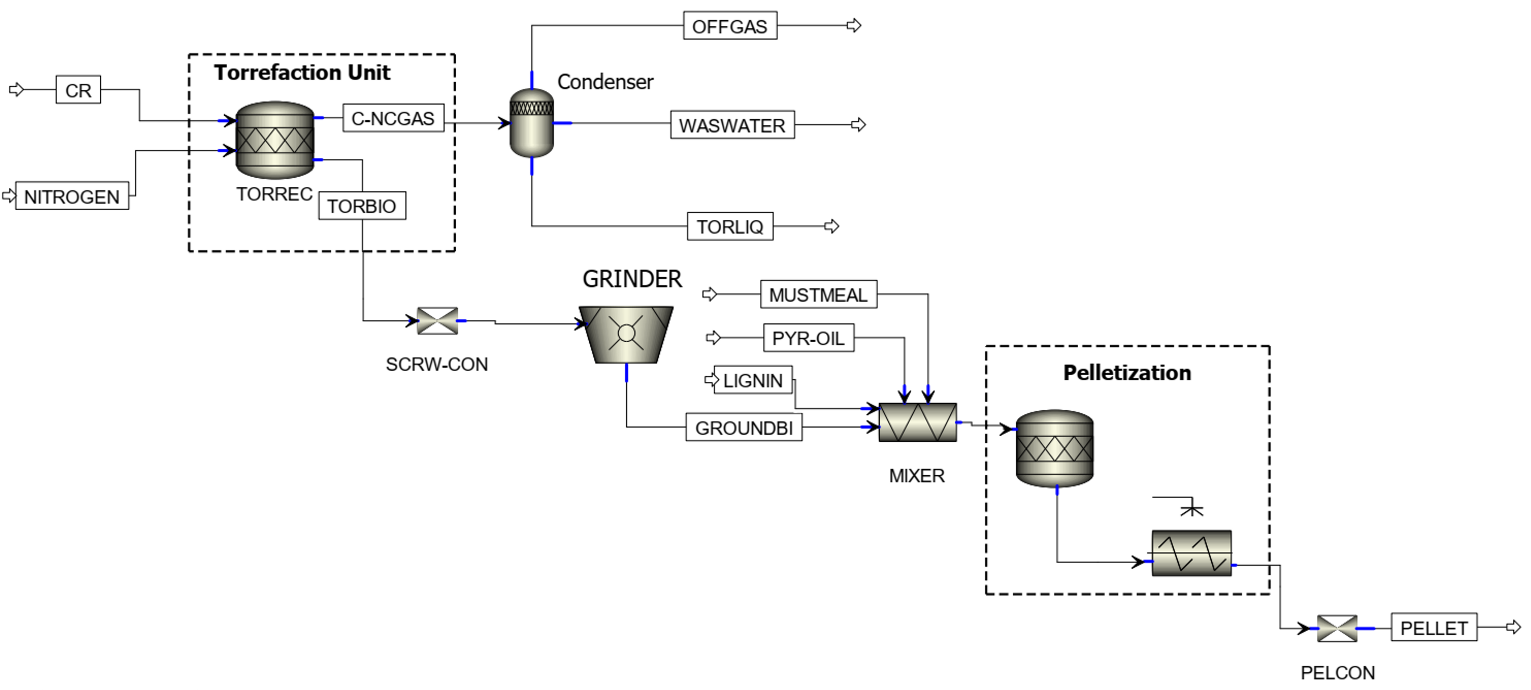


Fig. S1: Process flow diagram of integrated torrefaction and pelletization for torrefied pellet production with additives


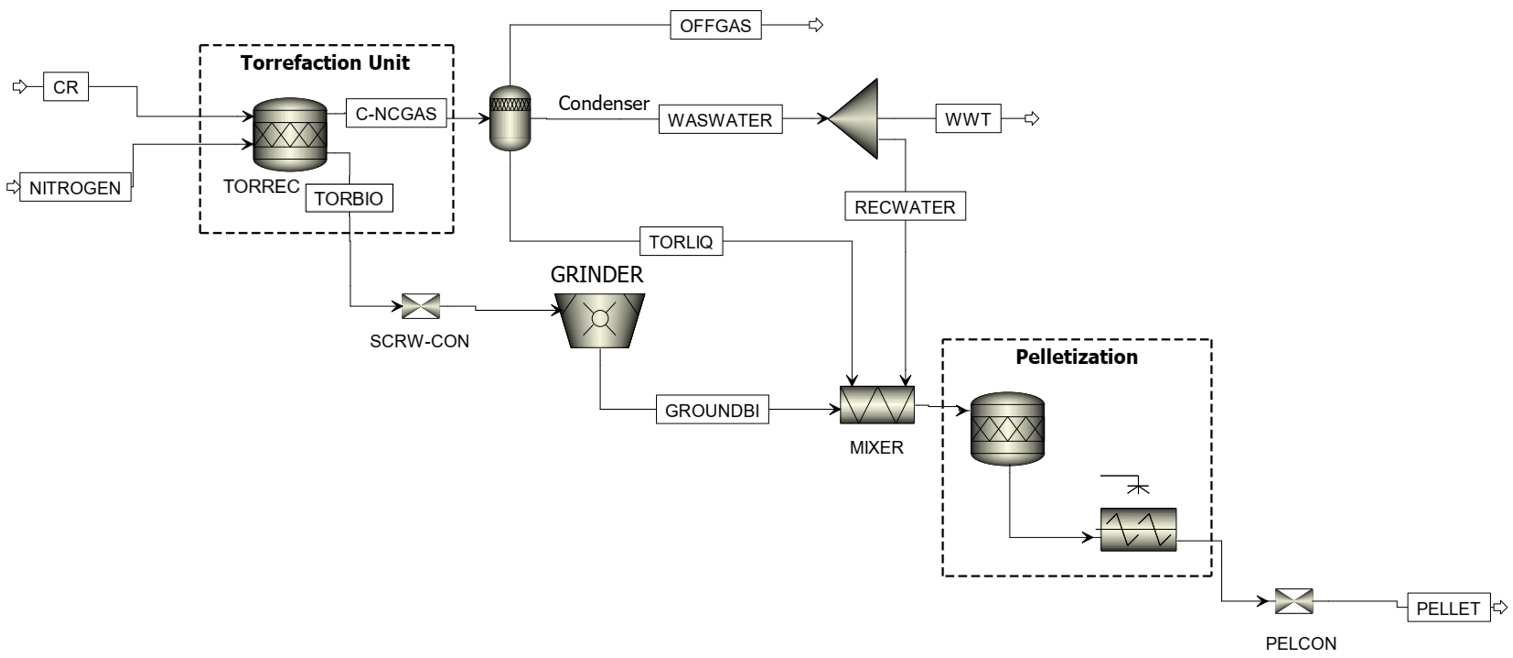


Fig. S2: Process flow diagram of integrated torrefaction and pelletization for torrefied pellet production without external additives

Table S1: Specification of unit operation used in simulation for torrefied pellet production

| Process unit | Code | Specifications |
| --- | --- | --- |
| Torrefaction | Rstoic Reactor | Temperature: 178 °C  Pressure: 1 bar |
| Cooler and conveyor | Screw Conveyor | Biomass in: 178 °C  Biomass out: 25°C  Length: 5 m |
| Grinder | crusher | Final particle size: 1.7 mm |
| Mixer | mixer | Pressure drop neglected |
| Pelletization | granulator | Temperature: 100 °C  Pressure: 100 MPa |
| Pellet cooler and conveyor | Vibratory conveyor | Pellet in: 100 °C  Pellet out: 25 °C  Length: 5 m |
| Condenser | Flash | Condenser and separator  Gravity separator  Temperature: 25 °C  Pressure: 1 bar |

Table S2: Total annual revenue cost estimation

| Items | Cost | |
| --- | --- | --- |
|  | Scenario 1 | Scenario 2 |
| Unit cost of torrefied pellet (tonne) | U.S. $170/tonne | U.S. $170/tonne |
| Annual production | 39898 tonne | 34196 |
| Total revenue from pellet | $6,782,660 | $5,813,320 |
| Total by-product credit | $879,601 | $0 |
| Waste-water cost | $0 | $0 |
| Total annual revenue | $7,662,261 | $5,813,320 |
